# Nucleation of protein aggregation kinetics as a basis for genotype-phenotype correlations in polyglutamine diseases

**DOI:** 10.1186/1750-1326-4-29

**Published:** 2009-07-15

**Authors:** Keizo Sugaya, Shiro Matsubara

**Affiliations:** 1 Department of Neurology, Tokyo Metropolitan Neurological Hospital, 2-6-1 Musashidai, Fuchu, Tokyo 183-0042, Japan

## Abstract

Recent studies of inherited neurodegenerative disorders have suggested a linkage between the propensity toward aggregation of mutant protein and disease onset. This is particularly apparent for polyglutamine (polyQ) diseases caused by expansion of CAG-trinucleotide repeats. However, a quantitative framework for relating aggregation kinetics with molecular mechanisms of neurodegeneration initiation is lacking. Here, using the repeat-length-dependent age-of-onset in polyQ diseases, we derived a mathematical model based on nucleation of aggregation kinetics to describe genotype-phenotype correlations, and validated the model using both *in vitro *data and clinical data. Instead of describing polyQ aggregation kinetics with a derivative equation, our model divided age-of-onset (equivalent to the time required for aggregation) into two processes: nucleation lag time (a first-order exponential function of CAG-repeat length) and elongation time. With the exception of spinocerebellar ataxia (SCA) 3, the relation between CAG-repeat length and age-of-onset in all examined polyQ diseases, including Huntington's disease, dentatorubral-pallidoluysian atrophy and SCA1, -2, -6 and -7, could be well explained by three parameters derived from linear regression analysis based on the nucleated growth polymerization model. These parameters composed of probability of nucleation at an individual repeat, a protein concentration associated factor, and elongation time predict the overall features of neurodegeneration initiation, including constant risk for cell death, toxic polyQ species, main pathological subcellular site and the contribution of cellular factors. Our model also presents an alternative therapeutic strategy according to the distinct subcellular loci by the finding that nuclear localization of soluble mutant protein monomers itself has great impact on disease onset.

## Background

Accumulation of misfolded proteins into protein aggregates is a hallmark of various aging-associated neurodegenerative diseases, including Alzheimer's, Parkinson's and polyglutamine (polyQ) diseases [1,2]. The biochemical properties of the affected proteins dictate their propensity to aggregate as well as the age-of-onset of these diseases. To date, nine inherited neurodegenerative disorders known as polyQ disease have been identified, including Huntington's disease (HD), spinal and bulbar muscular atrophy (SBMA), dentatorubral-pallidoluysian atrophy (DRPLA) and spinocerebellar ataxia (SCA) 1–3, -6, -7 and -17 [3]. These diseases have little in common at the genetic level other than the presence of polyQ sequence. However, there is a strong and consistent inverse relation between the length of the expansion and the age-of-onset in these disorders [3,4]. Despite the fact that an understanding of genotype-phenotype relationships might offer insights into the intrinsic toxicity of polyQ peptides and the contribution of tissue context factors, such correlational analyses have rarely been attempted for the various polyQ diseases [4-6]. Those studies that have been performed have provided limited information because the parameters derived from a simple exponential regression analysis are highly variable. Rather than providing evidence that the genotype-phenotype relationship is modified by the nature of the protein encoded by each disease gene, this variability suggests deficiencies in the basic model describing the relationship.

Another common feature of polyQ diseases is the neuronal accumulation of the mutant protein in nuclear or cytoplasmic inclusion [2,7]. In addition to the length dependence of disease onset, the length of polyQ sequence also predicted the propensity toward aggregation of polyQ-containing peptides [8,9]. The aggregation of polyQ peptides *in vitro *follows a simple nucleated growth polymerization pathway, implying crystallization or, in some cases, amyloid fibril formation [9,10]. Nucleated growth polymerization is a two-stage process consisting of the energetically unfavorable formation of a nucleus (i.e., nucleation), followed by efficient elongation of the nucleus via sequential additions of monomers [10]. Its kinetics is exemplified by long lag time followed by rapid aggregate growth, with a strong dependence of aggregation lag time on monomer concentration [10]. Nucleated growth polymerization has been proposed to govern disease progression kinetics in Alzheimer's and prion-related diseases [11]. We and others have previously suggested a linkage between the biophysics of polyQ aggregation nucleation and HD onset [9,12]. The actual mechanism of the generation of nuclei based on polyQ sequences will be structurally complex, but a kinetic parameter of nucleation is expected to be an exponential function of repeat length [13]. The polyQ length dependence of disease onset correlates strongly with the tendency of expanded polyQ proteins to aggregate in disease models [14,15]. Accordingly, we have focused on this length dependence of age-of-onset and nucleation kinetics to derive a stochastic mathematical model describing genotype-phenotype correlations in polyQ diseases.

Accumulating evidence strongly suggests that the cell nucleus is the main pathological subcellular site for SCA1, -7 and HD [16-18], whereas the cytoplasm is thought to be the site for SCA2 and SCA6 [19,20]. Our mathematical model clearly subdivided polyQ diseases into two groups in accordance with the presumed main pathological subcellular site. From a comparison of the parameters based on the nucleated growth polymerization model, our present study leads us to propose alternative therapeutic targets according to the distinct subcellular loci.

## Methods

### Data collection

Clinical data from affected patients with a mutation in the responsible gene were obtained from previous studies [5,6,21-37]. Patients with a homozygous mutation were excluded. An insufficient number of patients with SBMA and SCA17 were available, so these diseases were not included in the analysis. A total of 1398 patients were analyzed: 221 with SCA1, 141 with SCA2, 308 with SCA3, 132 with SCA6, 188 with SCA7, 317 with HD and 91 with DRPLA.

### Modeling

**Figure 1:**
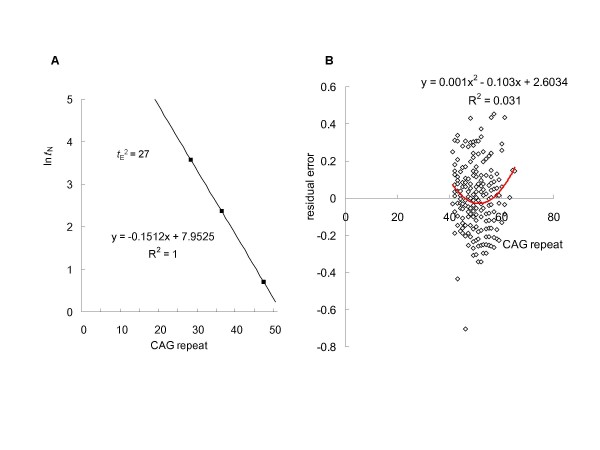
**Validity of the model using *in vitro *data and clinical data**. (**A**) The model was validated using *in vitro *data. It is impossible to experimentally determine the aggregation kinetics for polyQ peptides at physiological concentrations. However, assuming no change in mechanism, Chen *et al*, calculated the aggregation kinetics at low concentration (0.1 nM) from data obtained from aggregation of polyQ peptides at high concentration using the kinetics parameters of Equation 1 [9]. We tested the linear correlation of the natural log transform of these data, which represents the time required for 0.9% aggregation of polyQ peptides, against the polyQ tract number. A simple logarithmic transformation of the aggregation lag times did not show a linear relationship. In contrast, using *t*_*E*_^2 ^= 27 in our model, nucleation lag times perfectly matched a linear relation. (**B**) A linear regression analysis of natural log-transformed age-of-onset versus CAG size in SCA1 patients revealed that the plots of residual error versus CAG size were better fit to a U-shaped curve.

The derivation of equation describing polyQ peptide aggregation kinetics in a nucleated growth polymerization mechanism has been described by Chen *et al*. [9] as follows:

(1)

where Δ is the concentration of monomer that has been incorporated into polymers, *k*_+ _is the forward elongation rate constant, *k*_n* _is the equilibrium constant describing the monomer-nucleus equilibrium, *c *is the bulk concentration of monomers, n* is the critical nucleus, and *t *is time. This equation represents the overall pathway of nucleated growth polymerization. Further, kinetic studies also suggest that, in this mathematical model, the only factor related to CAG-repeat length is the nucleation constant, *k*_n*_[9,38,39]. However, nucleation kinetics cannot be determined directly through physical measurement of nuclei because nucleation is a very rare event and nuclei, once formed, quickly either collapse to bulk phase monomer or proceed along the productive aggregation pathway. Aggregation occurs with a lag phase and a growth phase that reflect an underlying nucleation-polymerization mechanism. The lag time of aggregation (aggregation lag time) as a kinetic parameter of nucleation was calculated by extrapolation of the linear region of the growth phase to the base line of the lag phase by experimental observation using Equation 1 (Figure 1A). However, polyQ aggregation kinetics also feature lag phases that can be abbreviated by seeding [8,40]. Instead of using polyQ aggregation kinetics to describe the overall pathway of nucleated growth polymerization, we hypothesized a mathematical model that divides the time required for aggregation into two processes: CAG-repeat-length-dependent nucleation lag time, and repeat-length-independent elongation time from elongation kinetics using aggregates of the polyQ peptides as a seed [8,38,39]. In this mathematical model, nucleation lag time (a proxy for aggregation lag time) is determined by the time required for aggregation and elongation time, as a function of repeat length.

In the nucleated growth polymerization pathway, aggregate growth is exponential. A previous study that employed a mutagenic analysis to the nucleation propensity of Alzheimer's β-amyloid showed that nucleation lag time of mutant β-amyloid could be related directly to the rate of aggregate growth [41]. Aggregate growth is largely dependent on nucleation lag time. In polyQ diseases, by contrast, the relationship between age-of-onset and the number of repeats is typically characterized by an exponential curve in which the change in the age-of-onset as a result of additional inherited repeats decreases with the number of repeats [4]. Moreover, as noted by Clarke *et al*., in clinically affected HD patients there is a first-order exponential decline in the number of surviving caudate nucleus neurons with time (based on metabolic activity), regardless of CAG-repeat length [42]. These findings, taken together with the linkage between aggregation nucleation of polyQ peptides and HD age-of-onset [9,12], suggest that the nucleation lag time for polyQ aggregation could be represented by a first-order exponential function of CAG-repeat lengths. *In vitro *studies also showed that the elongation rates of polyQ peptides changed little as a function of repeat-length [8,38,39]. Assuming such an identity in the elongation rate constant, and given the fact that the aggregation rate is *t*^2 ^dependent [9,39], the age-of-onset in polyQ diseases could be represented as a function of CAG-repeat lengths as follows:

(2)

where *t*_A _is age-of-onset of the disease (equivalent to the time required for aggregation), *t*_E _is elongation time, *t*_N _is nucleation lag time and *x *is the repeat-length number.

The identical relationship between nucleation lag time and CAG-repeat length was also proposed by Perutz et al. based on chemicalthermodynamics [13]. According to the theory of nucleation of aggregates, the probability of nucleation would be an exponential function of the free energy of formation of the nucleus, and is proportional to exp [-Δ*G*_*cri*t_/*kT*] [13]. Δ*G*_crit _is the critical free energy required to create a spherical nucleus, *k *is Boltzmann's constant, and *T *is absolute temperature. Since the addition of each glutamine stabilizes the helix structure by the formation of another three or four hydrogen bonds [43], alternation of free energy by the addition of each repeat to be expected constant. Therefore, probability of nucleation is likely to rise exponentially with the number of repeats. *P*_*n*+1_/*P*_*n *_= exp [Δ*G*_+1_/*kT*], where *P*_*n*+1 _is the probability of nucleation at repeat number *n *+ 1, *P*_*n *_is the probability of nucleation at repeat number n, Δ*G*_+1 _is the change of free energy by one additional repeat. Nucleation lag time is defined as that lag time which is required for the formation of a critical number of stable nuclei leading to polymerization. *In vitro *aggregation kinetics of polyQ peptide also showed that the critical nucleus – the number of monomeric units comprising the nucleus – is equal to 1 [9]. Thus, under the condition of monomeric nucleus, nucleation lag time could be represented by an exponential function of repeat length as follows:

(3)

where *N*_cri _is a critical number of nucleus leading to polymerization at a given space, *N*_A _is Avogadro's number, *C*_mo _is the bulk concentration of mutant protein monomers, *R*_0 _is the rate of formation of stable nuclei at the first structure, *m *is the number of repeat length at the first structure stable enough to form a nucleus, and e^*b *^is the probability of nucleation at an individual repeat.

### Statistical analysis

For statistical analysis, we used the UNISTAT 5.6 statistical package for Windows (Unistat). To examine the association between age-of-onset and CAG size, we used linear regression with logarithmic transformation, allowing an exponential function to be treated as an intrinsically linear model as follows:

(2a)

where In(*a*) is the intercept, *b *is the slope, and *ε *represents the residual error. Of course, the slope factor represents the probability of nucleation at an individual repeat and is determined by a nucleation constant, *k*_n*_, because *k*_n* _= c*/*c*^n^* (c* is the concentration of nuclei, *c *is the bulk concentration of monomers, n* is the critical nucleus) [10]. The intercept is composed of the complex factors (Equation 3). However, it is expected that its variation is mainly dependent on the concentration of soluble mutant protein monomers. A linear regression analysis was then applied to determine *t*_E_^2 ^for the best fit to a linear relationship between the logarithm of *t*_N _and CAG size. This was achieved by comparing the *R*^2 ^for a quadratic curve and a linear model. Then, to confirm that our mathematical model is a significantly better fit than a simple exponential regression model, we compared the *R*^2 ^values for linear regression analyses derived from simple natural log-transformed age-of-onset data without *t*_E_^2^. We used the residual error as another measure of goodness-of-fit. The small number of individuals with shortest and longest CAG-repeat sizes precluded a rigorous statistical analysis of these groups because they would have overruled the bulk of the other data and thus had too great an impact on the model. Applying this procedure, 94% to 98% of patients in each disease category were included in the analysis.

## Results

### Validated the model using *in vitro *data

In previous study, using the *in vitro *aggregation lag time of a series of polyQ peptides as a function of CAG size, described by Chen *et al*. [9], we compared the relationship between median age-at-onset of HD and particular repeat sizes, demonstrating a significant linear correlation [12]. Instead of using polyQ peptide aggregation kinetics to describe the overall pathway of nucleated growth polymerization [9], we successfully developed a mathematical model that divided the time required for aggregation (*t*_A_) into two processes governed by nucleation lag time (*t*_N_), a first-order exponential function of CAG size, and elongation time (*t*_E_), to yield the following relationship: (*t*_A_^2 ^- *t*_E_^2^)^1/2 ^= *t*_N _= *a*e^-*bx*^(see Methods). Then, we tested the linear correlation with logarithmic transformation, allowing an exponential function to be treated as an intrinsically linear model. A simple logarithmic transformation of the *in vitro *aggregation lag times against the polyQ tract number did not show a linear relationship (data not shown). These data represents the time required for 0.9% aggregation of polyQ peptides [9]. In contrast, when *t*_E_^2 ^= 27, the application of our model to *in vitro *nucleation lag times versus CAG size yields a plot that is perfectly fit with an exponential function (Figure 1A).

### Validated the model using clinical data

**Figure 2:**
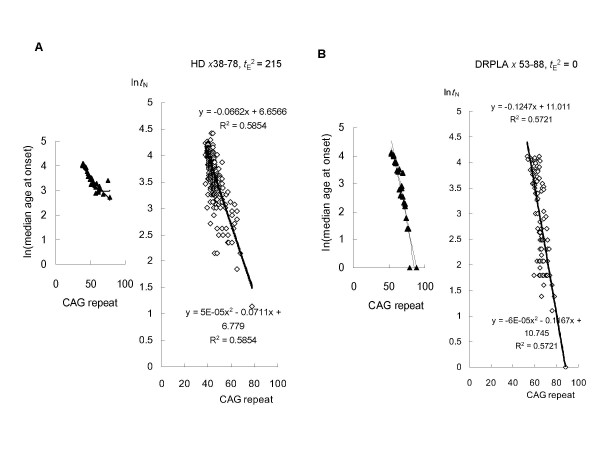
**Linear regression analysis of HD and DRPLA based on the model**. (**A**) A total of 317 HD patients with expanded CAG repeats in the huntingtin gene was analyzed. Inspection of logarithmically transformed mean age-at-onset versus CAG size plots identified 75 and 121 CAG as relative outliers. These data were therefore excluded from further study. A regression analysis of natural log-transformed (*t*_A_^2 ^- *t*_E_^2^)^1/2 ^against CAG size provided the best fit to a linear model (-0.0662*x *+ 6.657) when *t*_E_^2 ^= 215. (**B**) A total of 91 DRPLA patients with expanded CAG repeats in the atrophin-1 gene was analyzed. Inspection of logarithmically transformed mean age-at-onset versus CAG size plots identified 69 and 79 CAG repeats as relative outliers. These data were excluded from further study. A regression analysis of natural log-transformed (*t*_A_^2 ^- *t*_E_^2^)^1/2 ^against CAG size provided the best fit to a linear model (-0.1247*x *+ 11.011) when *t*_E_^2 ^= 0.

**Figure 3:**
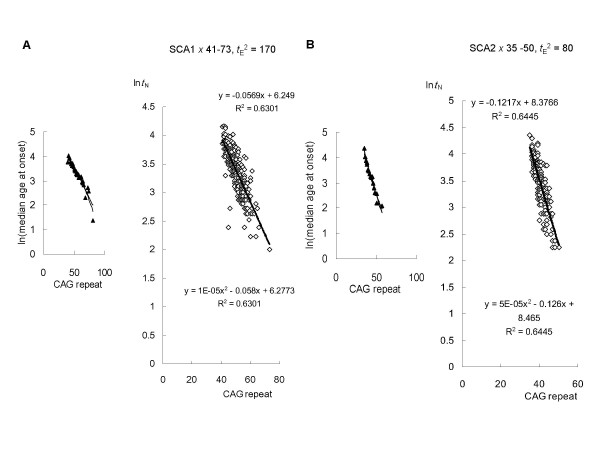
**Linear regression analysis of SCA1 and -2 based on the model**. (**A**) A total of 221 SCA1 patients with expanded CAG repeats in the ataxin-1 gene was analyzed. Inspection of logarithmically transformed mean age-at-onset versus CAG size plots identified 40, 69 and 81 CAG repeats as relative outliers. These data were therefore excluded from further analysis. Logarithmically transformed mean age-of-onset versus CAG size was the best fit to a quadratic curve. A regression analysis of natural log-transformed (*t*_A_^2 ^- *t*_E_^2^)^1/2 ^against CAG size provided the best fit to a linear model (-0.0569*x *+ 6.249) when *t*_E_^2 ^= 170. (**B**) A total of 141 SCA2 patients with expanded CAG repeats in the ataxin-2 gene was analyzed. Inspection of logarithmically transformed mean age-at-onset versus CAG size plots identified 51 and 57 CAG repeats as relative outliers. These data were excluded from further analysis. Logarithmically transformed mean age-of-onset versus CAG size was best fit to a quadratic curve. A regression analysis of natural log-transformed (*t*_A_^2 ^- *t*_E_^2^)^1/2 ^against CAG size provided the best fit to a linear model (-0.1217*x *+ 8.377) when *t*_E_^2 ^= 80.

**Figure 4:**
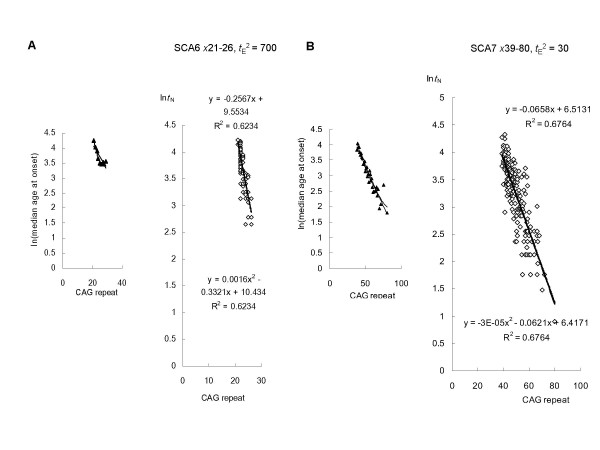
**Linear regression analysis of SCA6 and -7 based on the model**. (**A**) A total of 132 SCA6 patients with expanded CAG repeats in the alpha-1A-calcium channel gene was analyzed. Inspection of logarithmically transformed mean age-at-onset versus CAG size plots identified 27–29 CAG repeats as relative outliers and showed the best fit to a quadratic curve. A regression analysis of natural log-transformed (*t*_A_^2 ^- *t*_E_^2^)^1/2 ^against CAG size provided the best fit to a linear model (-0.2567*x *+ 9.553) when *t*_E_^2 ^= 700. (**B**) A total of 188 SCA7 patients with expanded CAG repeats in the ataxin-7 gene was analyzed. Inspection of logarithmically transformed mean age-at-onset versus CAG size plots identified 38 and 75 CAG repeats as relative outlier. These data were therefore excluded from further study. A regression analysis of natural log-transformed (*t*_A_^2 ^- *t*_E_^2^)^1/2 ^against CAG size provided the best fit to a linear model (-0.0658*x *+ 6.513) when *t*_E_^2 ^= 30.

Based on the nucleated growth polymerization model of polyQ peptides aggregation, we used the model for describing the relationship between CAG-repeat length and age-of-onset in polyQ diseases as follows: In(*t*_A_^2 ^- *t*_E_^2^)^1/2 ^= In(*a*) - *bx *+ *ε *(see Methods), where *t*_A _is age-of-onset of the disease (equivalent to the time required for aggregation), *ε *represents the residual error and *x *is the repeat-length number. We subsequently validated the model using available clinical data from patients with HD, DRPLA and SCA1–3, -6 and -7 [5,6,21-37]. A recent study of the relationship between age-of-onset in HD and CAG size in the HD gene has shown that a two-segment exponential regression curve provides a significantly better fit than a simple exponential regression model [44]. A similar tendency was also observed for SCA1, -2, -6, and -7. In fact, in these disorders, a linear regression analysis of natural log-transformed age-of-onset versus CAG size revealed that plots of residual error versus CAG size were consistently better fit to a U-shaped curve (Figure 1B). These findings strongly support our model, since a regression analysis of natural log-transformed age-of-onset, *t*_A _= (*t*_N_^2 ^+ *t*_E_^2^)^1/2^, versus CAG size is better fit to a U-shaped curve than a simple linear model, except for the case where elongation time is 0 (*t*_E _= 0). Therefore, elongation time is determined for each polyQ disease by identifying when the coefficients of determination (*R*^2^) for a quadratic curve and a linear model are identical (Figures 2, 3 and 4). This procedure restores the uncorrelation of the residual error, and consistently obtained higher *R*^2 ^values than linear regression analyses derived from simple natural log-transformed age-of-onset data without *t*_E_^2 ^in demonstrating a significant better fit than a simple exponential regression model (Table 1). We also analyzed the relationship using a logarithmic transformation of (*t*_A _- *t*) against CAG size, corresponding to an off-pathway of nucleated growth polymerization. Then, *t *is determined for each polyQ disease by identifying when the *R*^2 ^for a quadratic curve and linear model are identical (data not shown). However, a higher *R*^2 ^value was consistently obtained with (*t*_A_^2 ^- *t*_E_^2^)^1/2 ^versus CAG size, indicating an on-pathway of nucleated growth polymerization.

**Table 1:** Parameters based on the model: In (*t*_A_^2 ^- *t*_E_^2^)^1/2 ^= In(*a*) - *bx *+ *ε*

	HD	SCA1	SCA7	DRPLA	SCA2	SCA6
% patients	98.42	97.74	97.87	96.70	98.58	93.94
*t*_E_^2^	215	170	30	0	80	700
intercept (In(*α*))	6.657	6.249	6.513	11.011	8.377	9.553
± SE	0.1432	0.1516	0.1697	0.7543	0.3160	0.4076
slope (-*b*)	0.0662	0.0569	0.0658	0.1247	0.1217	0.2567
± SE	0.00316	0.00298	0.00338	0.0116	0.00773	0.0181
*F *test	437.65	364.59	380.43	114.96	248.33	201.92
(*P*)	3.23E-61	4.13E-48	1.84E-46	1.58E-17	1.45E-32	1.23E-27
*R*^2^	0.5854	0.6301	0.6764	0.5721	0.6445	0.6234
*R*^2 ^*	0.5321	0.6259	0.6595	0.5721	0.6391	0.6012

% patients = percent of patients comprised the model, SE = standard error, *R*^2 ^= coefficient determination, *R*^2 ^* = *R*^2 ^derived from linear regression analysis of simple logarithmic transformation of age-of-onset without *t*_E_^2^.

**Figure 5:**
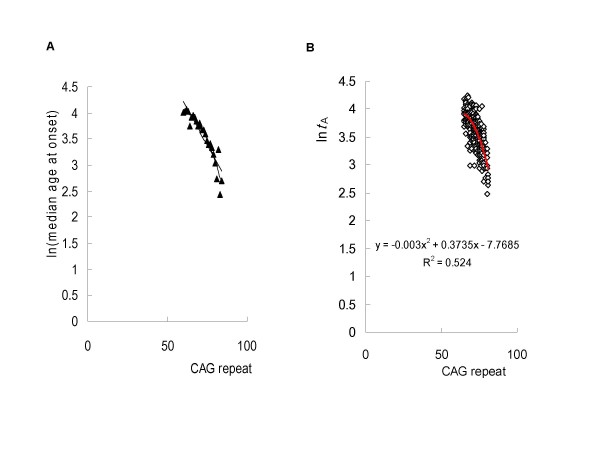
**Linear regression analysis of SCA3**. A total of 308 SCA3 patients with expanded CAG repeats in the ataxin-3 gene was analyzed. Inspection of logarithmically transformed mean age-at-onset versus CAG size plots (**A**) identified 64, 82 and 83 CAG repeats as relative outliers. However, in contrast to the other polyQ diseases, natural log-transformed age-of-onset versus CAG size was the best fit to a negative quadratic function (**B**). Clearly, this indicates that the nucleated growth polymerization model is unsuitable for SCA3.

In contrast to the linear regression analysis of genotype-phenotype correlation in other polyQ diseases we examined, SCA3 clearly did not conform to the nucleated growth polymerization model. A natural log-transformed age-of-onset in a total of 308 SCA3 patients versus CAG size in the ataxin-3 gene was the best fit to a negative quadratic function (Figure 5). This suggests that, despite shared pathological findings, including nuclear inclusion, a different mechanism underlies SCA3 pathogenesis.

The validity of the model is also supported by clinical data on disease progression. A statistical analysis of the detailed information provided by activity of daily living (ADL) milestones in SBMA patients and their relationship to the CAG-repeat length of the androgen receptor gene has demonstrated that the ADL milestone is significantly related to CAG size [45]. In contrast, the rate of disease progression showed no significant correlation to CAG size [45]. A similar lack of correlation between the rate of disease progression and CAG size was observed for HD patients [46]. Interestingly, the slopes of the regression curves for age-at-onset of each ADL milestone versus CAG size likely parallel one another [45], suggesting that the repeat-length-dependent parameter is conserved with respect to disease progression. In our model, the slope (*b*) factor is only dependent on CAG-repeat length, which is determined by a nucleation constant, *k*_n* _(see Methods). Therefore, the constancy of the slope could account for the fact that CAG-repeat length has little effect on the rate of disease progression, but is related to age-at-onset for each ADL milestone. In fact, the relationship between the age-at-onset of each ADL milestone in SBMA patients and CAG-repeat length also conformed to the nucleated growth polymerization model (data not shown).

### Linear regression analysis of HD, DRPLA, SCA1, -2, -6 and -7 based on the model

HD: A total of 317 HD patients with expanded CAG repeats in the huntingtin gene was analyzed. A regression analysis of natural log-transformed (*t*_A_^2 ^- *t*_E_^2^)^1/2 ^against CAG size provided the best fit to a linear model (-0.0662*x *+ 6.657) when *t*_E_^2 ^= 215 (Figure 2A). DRPLA: A total of 91 DRPLA patients with expanded CAG repeats in the atrophin-1 gene was analyzed. A regression analysis of natural log-transformed (*t*_A_^2 ^- *t*_E_^2^)^1/2 ^against CAG size provided the best fit to a linear model (-0.1247*x *+ 11.011) when *t*_E_^2 ^= 0 (Figure 2B). SCA1: A total of 221 SCA1 patients with expanded CAG repeats in the ataxin-1 gene was analyzed. A regression analysis of natural log-transformed (*t*_A_^2 ^- *t*_E_^2^)^1/2 ^against CAG size provided the best fit to a linear model (-0.0569*x *+ 6.249) when *t*_E_^2 ^= 170 (Figure 3A). SCA2: A total of 141 SCA2 patients with expanded CAG repeats in the ataxin-2 gene was analyzed. A regression analysis of natural log-transformed (*t*_A_^2 ^- *t*_E_^2^)^1/2 ^against CAG size provided the best fit to a linear model (-0.1217*x *+ 8.377) when *t*_E_^2 ^= 80 (Figure 3B). SCA6: A total of 132 SCA6 patients with expanded CAG repeats in the alpha-1A-calcium channel gene was analyzed. A regression analysis of natural log-transformed (*t*_A_^2 ^- *t*_E_^2^)^1/2 ^against CAG size provided the best fit to a linear model (-0.2567*x *+ 9.553) when *t*_E_^2 ^= 700 (Figure 4A). SCA7: A total of 188 SCA7 patients with expanded CAG repeats in the ataxin-7 gene was analyzed. A regression analysis of natural log-transformed (*t*_A_^2 ^- *t*_E_^2^)^1/2 ^against CAG size provided the best fit to a linear model (-0.0658*x *+ 6.513) when *t*_E_^2 ^= 30 (Figure 4B).

### Parameters derived from linear regression analysis

Three parameters, represented by the slope and intercept of the natural log-transformed linear regression curve, and elongation time, as well as descriptive statistics for each disease are summarized in Table 1. From Equations 3 (see Methods), the slope factor of the regression curve, e^*b*^, represents the probability of nucleation at an individual repeat (governed by the nucleation constant, *k*_n*_), and the intercept (In(*a*)) is interpreted as a variable that is inversely related to the concentration of soluble mutant protein monomers.

In genotype-phenotype correlations, each polyQ disease shows a characteristic threshold of CAG-repeat length [3,4]. SCA6 arises from a relatively small expansion with as few as 21 repeats. This contrasts with SCA1, -2, -7 and HD, where 35–40 repeats cause disease, and DRPLA, which arises from an even larger expansion (> 50 repeats). Three parameters derived from statistical analysis can well explain the relationship between CAG-repeat length and age-of-onset in any of these disorders. For example, these parameters indicate that a relatively large expansion required for disease onset in DRPLA is mainly due to the lower concentration of soluble mutant protein monomers (Figure 2B). In addition, a comparison of these parameters should reveal which factors strongly influence disease onset. In polyQ diseases, symptoms typically appear in adulthood; in DRPLA, infant-onset of the disease is occasionally seen, but this is never the case for SCA6. Our model clearly pointed to elongation time as a contributor to infant-onset, while adult-onset was mostly attributable to the nucleation lag time (Table 1).

### Variance in nucleation lag times

It is now believed that the identification of non-CAG size-dependent factors that explain residual onset age variance would possibly allow treatments that retard disease onset of polyQ disease. Regression analysis of HD, DRPLA and SCA1, -2, -6, and -7 showed that 57 to 68% variance of nucleation lag times is explained by the number of repeat units on the mutant allele (Table 1). However, using median nucleation lag time with a particular repeat size, there was a high significant correlation (*R *= 0.96 ~ 0.99) between CAG size and median nucleation lag times (data not shown), implying that the residual variance in nucleation lag times is mainly due to the variance at a particular repeat size. Our mathematical model (Equation 3) suggests that the concentration of soluble mutant protein monomers accounts for a major contributor to the residual variance in age-of-onset. Importantly, nucleation is a very rare event [13]. Therefore, only a small difference in concentration of soluble mutant protein monomers could result in a large difference in nucleation lag times, which directly reflects to onset age variance. In addition to the expression levels of a gene, the complexity of the cellular environment, including degradation and transport processes capable of partitioning proteins into different molecular forms and compartments, and the presence of chaperones will contribute to the differences in intracellular concentration of mutant protein.

## Discussion

Three parameters derived from linear regression analysis based on the nucleated growth polymerization model predict the overall features of neurodegeneration initiation of polyQ diseases, including toxic polyQ species, constant risk for cell death, main pathological subcellular site and the contribution of cellular factors, and provide an explanation for the aggregation of relatively short expanded polyQ tracts in SCA6.

Unexpectedly, a linear regression analysis of each disease showed that *t*_E_^2 ^varied from 0 to 700 (Table 1). This result, which is in contrast to the findings of a previous study [47], suggests that the specific oligomeric conformation of expanded polyQ is not uniformly toxic to neuronal cells in polyQ diseases. Instead, the validity of the nucleated growth polymerization model in any of these diseases indicates that toxic polyQ species accrues uniformly on-pathway of nucleated growth polymerization. Even though the disease-causing polyQ proteins are widely expressed, specific collections of neurons are more susceptible in each polyQ disease, resulting in characteristic patterns of pathology and clinical symptoms. Recent studies have suggested that altered protein function is fundamental to pathogenesis, with protein context of the expanded polyQ having key roles in disease specific processes [48]. Therefore, varied *t*_E _suggest that a growth phase of aggregation may represent disease specific processes affected by protein context of the expanded polyQ.

The clinical manifestations of inherited neurodegenerative diseases are often delayed for periods from years to decades. This observation has led to the idea that, in these disorders, neurons die from cumulative damage. Consistent with a cumulative damage model, a failure of protein folding quality control has been proposed for the pathogenesis of polyQ diseases [49,50]. A critical prediction of the cumulative damage hypothesis is that the probability of neuronal death increase over time (sigmoidal kinetics). However, Clarke *et al*. demonstrated that, in many aging-associated neurodegenerative diseases, including HD, the kinetics of neuronal death appear to be exponential [42,51]. Exponential kinetics, which also describe radioactive decay, indicates that in the neurodegenerations, the risk of cell death is constant (in some cases, as described by Clarke *et al*., an exponentially decreasing risk of death) over time. They accounted for this constancy by proposing a one-hit model in which the death of a neuron is initiated randomly in time by a single, rare catastrophic event rather than resulting from cumulative damage [42,51]. Thereafter, Perutz *et al*. proposed that nucleation is responsible for this kind of rare event, based on the fact that nucleation occurs randomly in time and space [13]. We have successfully established the common quantitative connection among polyQ diseases between the nucleation kinetics based on polyQ sequence and the repeat-length-dependent age-of-onset, implicating nucleation of protein aggregation kinetics as the basis for the genotype-phenotype correlations. In our model, the slope of the regression curve, which is a constant factor related only to CAG-repeat length, represents the probability of nucleation at an individual repeat and is equivalent to the constant risk of neuronal cells death at an individual repeat, consistent with Perutz's hypothesis [13]. Remarkably, nucleation and nucleation dependent polymerization exhibits kinetics consistent with both a constant and an exponentially decreasing risk of neuronal death in the neurodegenerations. Taken together, these findings provide indirect evidence that the genetic gain-of-function mechanism of polyQ pathogenesis is attributable to a critical nucleation event; thus, cytotoxicity accrues through the process of nucleation and nucleation-dependent polymerization.

In contrast to other polyQ diseases we examined, SCA3 does not fit our derived model (Figure 5), suggesting that another factor, dependent on the repeat-length of polyQ expansions, plays a critical role in determining disease onset of SCA3. Recent studies have suggested that ataxin-3 normally participates in protein quality control pathways in the cell [52,53]. Endoplasmic reticulum (ER)-associated degradation (ERAD) is a quality control system in the secretory pathway responsible for degrading misfolded proteins [54]. ERAD involves a series of steps to extract proteins from the ER and deliver them to proteasomes. The key protein essential for extracting substrates from the ER to the cytosol is valosin-containing protein [55]. It is of interest that ataxin-3 binds valosin-containing protein and regulates retrotranslocation of ERAD substrates in a repeat-length-dependent manner [53]. These findings underscore the critical role of protein quality control in SCA3 pathogenesis and raise a possibility that neuronal death in SCA3 exhibits sigmoidal kinetics.

Further study of disease progression as a function of CAG-repeat length for each polyQ disease will be needed to confirm the constancy of the slope and toxicity relationships. These analyses will more precisely characterize rates of disease progression whether it consists with exponential kinetics or sigmoidal kinetics.

One of the parameters, the slope of the regression curve, clearly subdivided polyQ diseases into two groups. One group included SCA1, -7 and HD; the other included SCA2, -6 and DRPLA (Table 1). Accumulating evidence strongly suggests that the cell nucleus is the main pathological subcellular site for SCA1, -7 and HD [16-18], whereas the cytoplasm is thought to be the site for SCA2 and SCA6 [19,20]. Clearly, the distribution into two groups is consistent with the presumed main pathological subcellular site in each disease. Further support for this idea is provided by the observation that a smaller intercept is consistently observed in the case of SCA1, -7 and HD (Table 1). Given the nucleus-to-cytoplasm volume ratio, it is expected that the concentration of degradation-resistant mutant proteins would be higher in the nucleus than in the cytoplasm. Another explanation for the smaller intercept in SCA1, -7 and HD might be related to the finding that the autophagy-lysosomal system is also involved in cytoplasmic degradation of aggregate-prone protein with polyQ, but the nucleus lacked this activity [56].

The slope of *in vitro *aggregation of polyQ peptides is 0.1512 (Figure 1A), which is close to the slope for SCA2 (0.1217). In contrast to conventional models of nucleated growth polymerization, the critical nucleus (the number of monomeric unit comprising the nucleus) for polyQ peptides aggregation is a monomer (*n *= 1) [9]. Assuming that the cytoplasmic pathological sites are identical, compared to the slope of SCA2, the slope of SCA6 (0.2567) corresponds to a dimeric nucleus (*n *= 2). To date, there are no data capable of providing a mechanism to explain the aggregation of such a short polyQ expansion in SCA6. Our model predicts that the aggregation in SCA6 is attributable to the dimeric nucleus, which promotes disease-onset about 20 times more effectively than a monomeric nucleus at a CAG size of 22. In contrast, the slopes of SCA1, -7 and HD are substantially lower than the value of 0.1512 (Table 1), which corresponds to the smallest critical nucleus size. Therefore, a certain inhibitory factor such as molecular chaperone or ubiquitin-proteasome system is inferred to exist in the cell nucleus that reduces the probability of nucleation to less than half the value of the slope. This is compatible with the idea that nuclear inclusion serves to protect against neurodegeneration in polyQ diseases [57]. Taken together with the finding that SCA3 operates by a different mechanism, this suggests that nuclear inclusion itself is an off-pathway product of nucleated growth polymerization.

**Figure 6:**
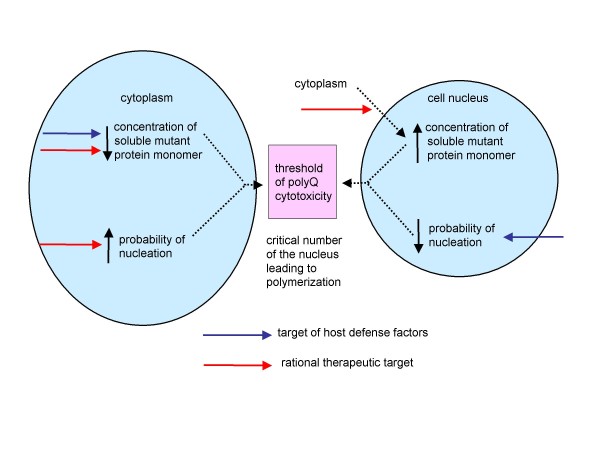
**Alternative therapeutic targets according to distinct subcellular loci**. The slope (probability of nucleation at an individual repeat) and intercept (inversely related to the concentration of soluble mutant protein monomers) determines a critical number of the nucleus leading to polymerization pathway, considering as a threshold of polyQ cytotoxicity. Host defense factors (blue arrow) considerably inhibit the probability of nucleation in the cell nucleus. By contrast, the concentration of soluble mutant protein monomers in the cell nucleus is quite higher than the values in the cytoplasm. A rational therapeutic target (red arrow) is to reduce the concentration of soluble mutant protein monomers in the cell nucleus, and is to inhibit the probability of nucleation and reduce the concentration of soluble mutant protein monomers in the cytoplasm.

Based on the nucleated growth polymerization model, the parameters derived from linear regression analysis have led us to propose alternative therapeutic targets according to the distinct subcellular loci (Figure 6). The slope (probability of nucleation at an individual repeat) and intercept (inversely related to the concentration of soluble mutant protein monomers) determines a critical number of the nucleus, which initiates polymerization pathway, considered as a threshold of polyQ cytotoxicity. Our results suggest that, in the cell nucleus, host defense factors considerably inhibit the probability of nucleation at an individual mutant protein. By contrast, it is estimated that the concentration of soluble mutant protein monomers in the cell nucleus is quite higher than the values in the cytoplasm (e^8.377~11.011 ^versus e^6.249~6.657^, the mean ratio is about 40:1). Therefore, nuclear localization of the mutant protein itself has great impact on the age-of-onset in SCA1, -7 and HD. Theoretically, an attractive therapeutic target is to reduce the concentration of soluble mutant protein monomers in the cell nucleus, particularly by inhibition of translocation of the mutant proteins from the cytoplasm to the cell nucleus, and thereby delay polyQ nucleation aggregation.

## Conclusion

One of the striking findings of neurodegeneration research is the observation that most of the proteins implicated in disease have a strong propensity to aggregate. Aggregation is a central aspect of the biology of many neurodegenerative diseases. However, the role of aggregates in neurodegeneration is unclear. In this article, we present the first development of a mathematical model, which describes the basis for genotype-phenotype correlations in inherited neurodegenerative disorders known as polyQ disease and have successfully established the common quantitative connection among polyQ diseases between the repeat-length-dependent age-of-onset and aggregation kinetics based on polyQ sequence. Our results have clear implications for polyQ disease pathogenesis and therapy.

## Competing interests

The authors declare that they have no competing interests.

## Authors' contributions

Conceived and designed the experiments: KS. Analyzed the data: KS. Wrote the paper: KS SM. All authors read and approved the final manuscript.
